# Fibrinogen is a promising biomarker for chronic obstructive pulmonary disease: evidence from a meta-analysis

**DOI:** 10.1042/BSR20193542

**Published:** 2020-07-24

**Authors:** Bo Zhou, Shufang Liu, Danni He, Kundi Wang, Yunfeng Wang, Ting Yang, Qi Zhang, Zhixin Zhang, Wenquan Niu

**Affiliations:** 1 Graduate School, Beijing University of Chinese Medicine, Beijing, China; 2 Department of Pediatrics, China-Japan Friendship Hospital, Beijing, China; 3 Institute of Clinical Medical Sciences, China-Japan Friendship Hospital, Beijing, China; 4 National Clinical Research Center for Respiratory Diseases, Beijing, China; 5 Department of Pulmonary and Critical Care Medicine, China-Japan Friendship Hospital, Beijing, China; 6 Clinical Diagnosis Department of Respiratory Diseases Center, China-Japan Friendship Hospital, Beijing, China; 7 International Medical Services, China–Japan Friendship Hospital, Beijing, China

**Keywords:** Chronic obstructive pulmonary disease, Fibrinogen, Meta-analysis, Risk, Severity

## Abstract

**Backgrounds:** Some studies have reported association of circulating fibrinogen with the risk of chronic obstructive pulmonary disease (COPD), and the results are conflicting. To yield more information, we aimed to test the hypothesis that circulating fibrinogen is a promising biomarker for COPD by a meta-analysis.

**Methods:** Data extraction and quality assessment were independently completed by two authors. Effect-size estimates are expressed as weighted mean difference (WMD) with 95% confidence interval (95% CI).

**Results:** Forty-five articles involving 5586/18604 COPD patients/controls were incorporated. Overall analyses revealed significantly higher concentrations of circulating fibrinogen in COPD patients than in controls (WMD: 84.67 mg/dl; 95% CI: 64.24–105.10). Subgroup analyses by COPD course showed that the degree of increased circulating fibrinogen in patients with acute exacerbations of COPD (AECOPD) relative to controls (WMD: 182.59 mg/dl; 95% CI: 115.93–249.25) tripled when compared in patients with stable COPD (WMD: 56.12 mg/dl; 95% CI: 34.56–77.67). By COPD severity, there was a graded increase in fibrinogen with the increased severity of COPD relative to controls (Global Initiative for Obstructive Lung Disease (GOLD) I, II, III, and IV: WMD: 13.91, 29.19, 56.81, and 197.42 mg/dl; 95% CI: 7.70–20.11, 17.43–40.94, 39.20–74.41, and −7.88 to 402.73, respectively). There was a low probability of publication bias.

**Conclusion:** Our findings indicate a graded, concentration-dependent, significant relation between higher circulating fibrinogen and more severity of COPD.

## Introduction

Chronic obstructive pulmonary disease (COPD) is an escalating public health problem that affected more than 174.5 million adults in 2015 worldwide [1]. At present, COPD ranks as the third leading cause of mortality [2], and is responsible for approximately 3 million deaths annually [2,3]. Given that COPD can impair the quality of life by causing progressive reduction in lung function, loss of exercise capacity, increase in hospital admissions, and premature mortality [4]. COPD prevention represents an important public health goal. Because COPD is a progressive lung disease, the identification of biomarkers for early detection of COPD may help to improve future respiratory health.

It is widely recognized that systemic inflammation is an important clinical feature of COPD [5,6]. Much attention has been focused on the potential implication of circulating inflammatory biomarkers in the development of COPD. One of the most widely evaluated inflammatory biomarkers is fibrinogen, a key modulator of inflammation and fibrosis development, as well as tissue injury [7]. The association between circulating fibrinogen and COPD risk has been investigated by a large number of studies, with inconsistent and inconclusive findings. For example, some researchers have reported a significantly higher concentration of circulating fibrinogen in COPD patients than healthy controls [8–10], whereas others found that circulating fibrinogen concentration was comparable between the two groups [11], and even a significantly higher concentration in controls [12,13]. In a recent umbrella review of meta-analyses, Bellou and colleagues synthesized observational data on environmental factors and biomarkers in possible association with COPD, and found that circulating fibrinogen was a promising clinical marker of COPD, yet with very large heterogeneity [14]. Despite the significant heterogeneity, plasma fibrinogen this year has been qualified as a COPD biomarker for severity assessment in the United States by the Food and Drug Administration (FDA) [15]. Kirkpatrick and Dransfield have written an excellent review, and demonstrated that race may influence COPD susceptibility and progression [16]. It is hence reasonable to speculate that such very high heterogeneity is likely attributed to racial differences. Besides, other possible reasons such as different study designs and individually underpowered studies also account for this issue. However, the reasons behind inconsistence and heterogeneity when assessing the relationship between circulating fibrinogen and COPD thus far remains unexplored.

To fill this gap in our knowledge and generate more information for future studies, we prepared a comprehensive meta-analysis of published observational studies to test the hypothesis that circulating fibrinogen is a promising biomarker for COPD, and if this hypothesis is confirmed, we further examined whether circulating fibrinogen is associated with the severity of COPD in a concentration-dependent fashion.

## Methods

We conducted this meta-analysis of observational studies in compliance with the requirements of the Preferred Reporting Items for Systematic reviews and Meta-Analyses (PRISMA) statement [17]. The PRISMA checklist is provided in Supplementary Table S1.

### Search strategy

A systematic electronic search of PubMed, EMBASE (Excerpt Medica Database), Cochrane Central Register of Controlled Trials, and Web of Science was conducted from inception to 25 June 2019 for publications that assessed the relationship between circulating fibrinogen and COPD. The key terms used for search included ‘fibrinogen’, ‘FIB’, ‘chronic obstructive pulmonary disease’, and ‘COPD’.

Literature search was restricted to articles published in the English language from peer-reviewed journals. Additional articles were obtained via manually scanning the reference lists of relevant reviews and major original articles. Literature search was independently completed by two authors (B.Z. and W.N.), and divergences were resolved by discussion until a consensus was reached.

### Eligibility criteria

Articles were included if they were observational case–control studies, and if they had mean or median values of circulating fibrinogen concentration in both COPD patients and controls, along with standard deviation or standard error or interquartile range or whole range. Articles were excluded if they lacked control groups, or if they published in form of reviews, case reports, case series, conference abstracts, letter to the editor, comments, or editorials.

### Article selection

Two authors (B.Z. and W.N.) independently screened the titles and abstracts of all retrieved articles, and if necessary the full texts to assess their eligibility. If more than one article was published from the same or part of study participants, the article with the largest sample size was retained in the analysis. Any disagreement was discussed, and when necessary, adjudicated by a third author (Z.Z.).

### Data extraction

Data from qualified articles were independently extracted by two authors (B.Z. and W.N.), including surname of first author, year of publication, country where the study was conducted, sample size, study type, COPD and its stages, circulating concentrations of fibrinogen in both patients and controls, and necessary baseline characteristics, if available. Extracted data from the two authors were checked for consistency using κ statistic, and any divergences were resolved by a third author (Z.Z.).

### Quality assessment

The quality assessment tool, the 9-star Newcastle–Ottawa Scale, was employed [18]. This scale ranges from 0 (the worst) to 9 stars (the best). The following three major study components were judged: selection (0–4 stars), comparability (0–2 stars), and exposure/outcome (0–3 stars).

### Statistical analyses

The STATA software Release 14.1 (Stata Corp, College Station, TX) was used for statistical analyses. The difference in circulating fibrinogen concentration between COPD patients and healthy controls is expressed as weighted mean difference (WMD) and 95% confidence interval (95% CI) under the random-effects model using the DerSimonian and Laird method [19]. Heterogeneity is judged by the χ^2^ test and quantified by the inconsistency index (*I*^2^) statistic, which ranges from 0 to 100%. Heterogeneity is statistically significant if the probability of χ^2^ test is less than 10% or *I*^2^ is over 50%. To explore potential sources of heterogeneity, subgroup analyses were conducted according to COPD course (stable and acute exacerbations), COPD severity (Global Initiative for Obstructive Lung Disease (GOLD) I, GOLD II, GOLD III, and GOLD IV), smoking habit in controls, region, sample size and study type, and meta-regression analyses were further conducted to assess the confounding impact of age and gender.

Cumulative analyses were done to assess the impact of first published study on subsequent studies and evolution of accumulated estimates over time. Influential analyses were used to assess the contribution of single study to overall estimate.

Publication bias was evaluated by the Begg’s funnel plots and the Egger’s tests at a significance level of 10% [20]. The trim and fill method was used to predict the number of potentially missing studies and derive the ‘unbiased’ estimates.

## Results

### Eligible articles

**Figure 1:**
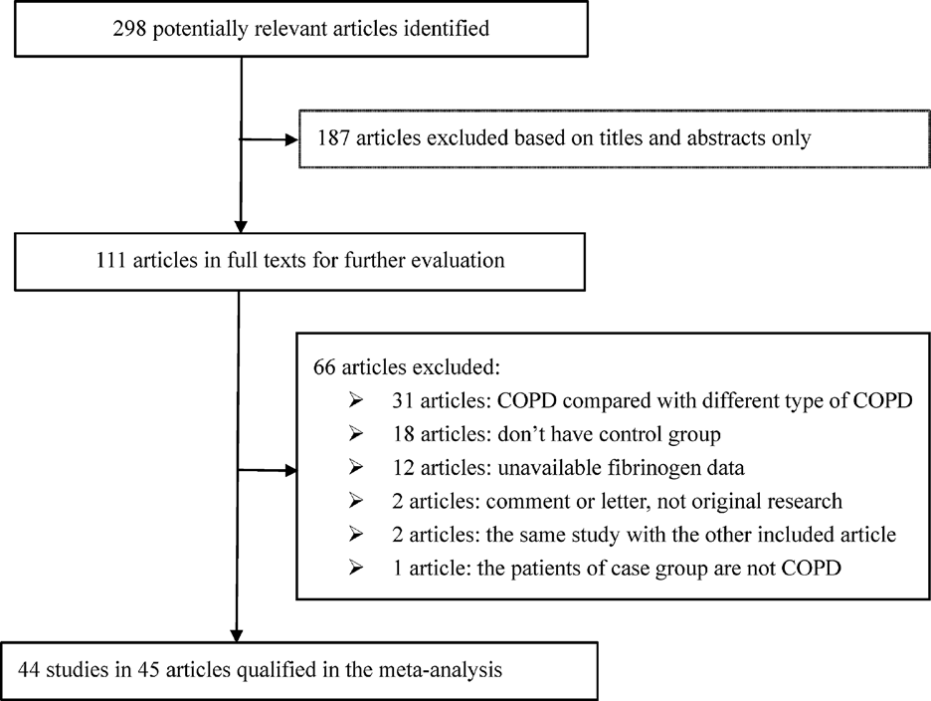
Flow diagram illustrating the selection of qualified studies with specific reasons for exclusion

In total, 298 potentially relevant articles were identified after literature search for observational studies on circulating fibrinogen and COPD. On the basis of titles and abstracts, 187 articles were excluded with obvious reasons, leaving 111 articles for further evaluation in full texts. Finally, there were 45 articles in this meta-analysis [8–13,21–59], involving 5586 COPD patients and 18604 controls, and all articles were published from the year 1997 to 2019. Because two articles respectively provided data in COPD patients as a whole [46] and by COPD severity (stable and acute exacerbations) [9] based on the same study participants, we combined results from the two articles as a single study. So, a total of 44 studies were synthesized in this meta-analysis. The specific reasons for exclusion during article selection are provided in Figure 1.

### Characteristics of qualified studies

Shown in Table 1 are the characteristics of all qualified studies. There were 14 studies enrolling patients with stable COPD, 11 studies with COPD exacerbations, and 9 studies with different COPD stages according to the GOLD guidelines. Eleven studies enrolled smokers as controls, and ten studies enrolled non-smokers as controls. The mean or median age of study participants ranged from 55 to 75 years. The sample size ranged from 16 to 1755.

**Table 1:** The baseline characteristics of qualified studies in this meta-analysis

First author	Year	Region	Patients	Controls	Sample size	Gender (M/F)	Age (years)	Smoke pack-years	Fibrinogen (mg/dl) in patients	Fibrinogen (mg/dl) in controls
Ronnow	2019	Europe	COPD	Smokers	95/95	49/49	60/59.4	38/30	356.4 ± 75.85	343.4 ± 53.78
Ronnow	2019	Europe	COPD	Non-smokers	95/95	49/49	60/58.8	38/0	356.4 ± 75.85	326.6 ± 65.3
Olloquequi	2019	America	COPD	Healthy	49/52	29/71	69.41/70.34	41.57/0	392.22 ± 106.38	319.81 ± 70.52
Jin	2018	Asia	COPD	Healthy	134/125	39.6/45.6	64.5/63.8	32/31.6	540 ± 80	250 ± 50
Aleva	2018	Europe	Stable COPD	Healthy	30/25	53.7/64	61.6/53	50.7/1.6	331.4 ± 141.9	305.1 ± 122.5
AboEI-Magd	2018	Africa	AECOPD	Healthy	45/20	31.1/35	57.71/56.65	39.62/31	567.3 ± 216.6	315.38 ± 68.18
AboEI-Magd	2018	Africa	GOLD I/II	Healthy	23/20	31.1/35	57.71/56.65	39.62/31	443.47 ± 107.98	315.38 ± 68.18
AboEI-Magd	2018	Africa	GOLD III	Healthy	13/20	31.1/35	57.71/56.65	39.62/31	595.38 ± 229.98	315.38 ± 68.18
AboEI-Magd	2018	Africa	GOLD IV	Healthy	9/20	31.1/35	57.71/56.65	39.62/31	843.33 ± 125	315.38 ± 68.18
Zeng	2017	Asia	COPD	Healthy	106/106	12.26/12.26	69.48/69.27	NA	429 ± 160	250 ± 54
Ugurlu	2017	Asia	AECOPD	Healthy	16/12	NA	NA	NA	446.44 ± 193.65	321.18 ± 115.24
Ugurlu	2017	Asia	Stable COPD	Healthy	13/12	NA	NA	NA	292.27 ± 74.51	321.18 ± 115.24
Lopez-Sanchez	2017	Europe	Stable COPD	Healthy	35/11	8.6/18.2	66.3/65.4	50/10	490 ± 133.3	310 ± 37.04
Golpe	2017	Europe	COPD	Healthy	20/20	25/25	70.1/NA	62.8/NA	387 ± 88.15	328 ± 40.74
Diao	2017	Asia	Stable COPD	Smokers	53/33	NA	64/58	38/31	330 ± 80	270 ± 70
Diao	2017	Asia	GOLD I	Smokers	10/33	NA	66/58	39/31	310 ± 54	270 ± 70
Diao	2017	Asia	GOLD II	Smokers	15/33	NA	63/58	38/31	330 ± 100	270 ± 70
Diao	2017	Asia	GOLD III	Smokers	19/33	NA	64/58	38/31	350 ± 66	270 ± 70
Diao	2017	Asia	GOLD IV	Smokers	9/33	NA	62/58	39/31	360 ± 100	270 ± 70
Arellano-Orden	2017	Europe	COPD	Smokers	96/33	0/0	67/58	71.9/46.9	520.5 ± 126.4	595 ± 133.3
Zhang	2016	Asia	Stable COPD	Healthy	43/43	20.9/23.3	62.3/60.8	47.3/43.9	297 ± 34.3	271 ± 66.8
Zhang	2016	Asia	AECOPD	Healthy	43/43	20.9/23.3	62.3/60.8	47.3/43.9	352 ± 81.3	271 ± 66.8
Golpe	2016	Europe	COPD	Smokers	67/67	22.4/22.4	59.4/58.2	52.7/43.7	378.4 ± 69.6	352.2 ± 45.6
Akiki	2016	Asia	COPD	Healthy	90/180	42.2/63.5	62/55	NA	299.2 ± 104.8	313.5 ± 64.2
Tudorache	2015	Europe	Stable COPD	Healthy	22/20	NA	63/63	NA	303 ± 70	262 ± 40
Tudorache	2015	Europe	AECOPD	Healthy	19/20	NA	63/63	NA	584 ± 62	262 ± 40
Stoll	2015	Europe	COPD	Smokers	54/21	38.9/38.1	59/61	38/39	420 ± 105	360 ± 72.5
Stoll	2015	Europe	COPD	Non-smokers	54/21	38.9/42.9	59/63	38/0	420 ± 105	300 ± 60
Mutlu	2015	Asia	Stable COPD	Healthy	29/29	16/10	68/64	NA	366 ± 128	345 ± 174
Mutlu	2015	Asia	AECOPD	Healthy	29/29	16/10	65/64	NA	487 ± 245	345 ± 174
Ishikawa	2015	Asia	COPD	Smokers	47/30	4.3/6.7	70.8/62.7	62.3/57.3	300 ± 100	303.5 ± 101.2
Ishikawa	2015	Asia	COPD	Non-smokers	47/20	4.3/50	70.8/59	62.3/0.34	300 ± 100	289 ± 96.3
Gumus	2015	Asia	AECOPD	Healthy	43/30	7/17	68/64	53/40	581.4 ± 353.7	237.8 ± 207.4
Boyuk	2015	Asia	COPD	Non-smokers	43/38	44.2/NA	NA	NA	363.53 ± 93.36	356 ± 50.33
Boyuk	2015	Asia	GOLD I	Non-smokers	9/38	NA	57.67/NA	NA	361.89 ± 72.63	356 ± 50.33
Boyuk	2015	Asia	GOLD II	Non-smokers	21/38	NA	65.14/NA	NA	371.1 ± 92.41	356 ± 50.33
Boyuk	2015	Asia	GOLD III	Non-smokers	13/38	NA	60.23/NA	NA	352.46 ± 111.91	356 ± 50.33
Pizarro	2014	Europe	COPD	Smokers	62/17	6/29	62/59	60/41	400 ± 96.3	390 ± 66.7
Pizarro	2014	Europe	COPD	Non-smokers	62/18	6/61	62/58	60/0	400 ± 96.3	330 ± 74.1
Gagnon	2014	America	GOLD I	Healthy	37/19	32/31	65/62	44/36	234 ± 63	345 ± 249
Can	2014	Asia	Stable COPD	Healthy	46/41	13/26.8	55.92/52.41	39.63/5.56	406.77 ± 172.6	336.53 ± 96.1
Can	2014	Asia	GOLD II	Healthy	17/41	13/26.8	55.92/52.41	39.63/5.56	354.06 ± 170.5	336.53 ± 96.1
Can	2014	Asia	GOLD III	Healthy	15/41	13/26.8	55.92/52.41	39.63/5.56	397.55 ± 162.1	336.53 ± 96.1
Can	2014	Asia	GOLD IV	Healthy	14/41	13/26.8	55.92/52.41	39.63/5.56	480.81 ± 192.7	336.53 ± 96.1
Wang	2013	Asia	Stable COPD	Healthy	70/70	14.3/18.6	69/68	56.4/54.3	417.7 ± 91.4	366.7 ± 101.4
Wang	2013	Asia	AECOPD	Healthy	70/70	14.3/18.6	69/68	56.4/54.3	466.1 ± 90.3	366.7 ± 101.4
Lazzeri	2013	Europe	COPD	Healthy	71/747	33.8/26.8	74/67	54/46.1	434 ± 148.9	391 ± 98.5
Waschki	2012	Europe	COPD	Smokers	127/22	39.20/45.5	64.6/68	38.3/27.2	504 ± 122	455 ± 129
Waschki	2012	Europe	COPD	Non-smokers	127/22	39.20/68.2	64.6/66.4	38.3/0	504 ± 122	408 ± 57
Lazovic	2012	Europe	COPD	Healthy	43/40	39.50/47.5	61.8/45.45	28.2/5.94	603 ± 229	414 ± 163
Gopal	2012	Europe	Stable COPD	Healthy	146/81	45.90/44.4	59.56/58.78	36.37/35.41	351.8 ± 84.5	330 ± 30
Cockayne	2012	America	GOLD I/II	Smokers	75/15	28/27	66.9/66.8	51.9/42.9	430 ± 74.1	460 ± 37
Cockayne	2012	America	GOLD III/IV	Non-smokers	65/30	25/30	66.3/66.4	58.8/0.5	460 ± 81.5	370 ± 59.3
Agusti	2012	Europe	COPD	Smokers	1755/297	34/45	63.5/55.5	48.9/31.7	448 ± 95.6	391 ± 65.2
Agusti	2012	Europe	COPD	Non-smokers	1755/202	34/62	63.5/53	48.9/0.2	448 ± 95.6	369 ± 78.5
Valvi	2012	America	GOLD I	Healthy	2669/7271	NA	NA	NA	309.5 ± 67.16	294.6 ± 59.69
Valvi	2012	America	GOLD II	Healthy	2221/7271	NA	NA	NA	320.7 ± 70.69	294.6 ± 59.69
Valvi	2012	America	GOLD III/IV	Healthy	585/7271	NA	NA	NA	336.8 ± 74.98	294.6 ± 59.69
Dickens	2011	Europe	COPD	Smokers	201/37	27/32	64.5/60.7	45.7/29.3	466 ± 117.5	425 ± 100
Dickens	2011	Europe	COPD	Non-smokers	201/37	27/62	64.5/60	45.7/0	466 ± 117.5	387 ± 83
Dickens	2011	Europe	Stable COPD	Smokers	157/37	NA	NA	NA	464 ± 115	425 ± 100
Dickens	2011	Europe	AECOPD	Non-smokers	33/37	NA	NA	NA	534 ± 156	387 ± 83
Selcuk	2010	Asia	COPD	Healthy	85/39	40/41	58.4/57.8	28/22	332 ± 129	295 ± 73
Garcia-Rio	2010	Europe	COPD	Healthy	324/110	26/54	64/55	40/10	347.8 ± 97.5	312.3 ± 71.8
Garcia-Rio	2010	Europe	GOLD I	Healthy	177/110	32.2/54	62/55	30/10	346 ± 104	312.3 ± 71.8
Garcia-Rio	2010	Europe	GOLD II	Healthy	128/110	18/54	67/55	45/10	363 ± 111	312.3 ± 71.8
Garcia-Rio	2010	Europe	GOLD III	Healthy	19/110	15.8/54	70/55	40/10	373 ± 117	312.3 ± 71.8
Yanbaeva	2009	Europe	COPD	Healthy	355/195	38/52	64.2/54.3	39.9/29.6	360 ± 43.7	330 ± 44.4
Watz	2009	Europe	GOLD I	Healthy	34/30	26/23	66.3/62.6	46.9/53.2	395 ± 64	410 ± 81
Watz	2009	Europe	GOLD II	Healthy	57/30	28/23	63.3/62.6	50.7/53.2	431 ± 98	410 ± 81
Watz	2009	Europe	GOLD III	Healthy	43/30	19/23	63.3/62.6	55.6/53.2	468 ± 115	410 ± 81
Watz	2009	Europe	GOLD IV	Healthy	36/30	25/23	63.7/62.6	54/53.2	444 ± 89	410 ± 81
Undas	2009	Europe	Stable COPD	Healthy	56/56	8.9/14.3	64.9/63.8	NA	408 ± 160	271 ± 57
Valipour	2008	Europe	Stable COPD	Healthy	30/30	30/30	60/59	58/27	424 ± 74.8	360 ± 49.6
Valipour	2008	Europe	AECOPD	Healthy	30/30	23/30	62/59	57/27	419 ± 104.4	360 ± 49.6
Polatli	2008	Asia	Stable COPD	Healthy	33/16	NA	63.42/59.63	33.64/21.56	346.88 ± 92.3	289.99 ± 39.9
Polatli	2008	Asia	AECOPD	Healthy	26/16	NA	68/59.63	45.04/21.56	447.67 ± 128	289.99 ± 39.9
Kunter	2008	Asia	AECOPD	Non-smokers	30/10	13.3/NA	72.37/64.5	NA	623.77 ± 189.45	305.7 ± 77.73
Higashimoto	2008	Asia	COPD	Healthy	111/75	2.7/2.7	74.9/64.5	61/41.2	340 ± 126.4	322 ± 103.9
Eickhoff	2008	Europe	COPD	Smokers	60/20	45/60	62/59	66/39	426 ± 87.4	367 ± 51.1
Eickhoff	2008	Europe	COPD	Non-smokers	60/20	45/65	62/62	66/0	426 ± 87.4	382 ± 82.2
Dentener	2008	Europe	COPD	Healthy	16/25	37.5/52	62/56	28/15	244 ± 56	182 ± 30
Mannino	2003	America	GOLD I	Healthy	1260/8446	NA	NA	NA	292 ± 51	281 ± 47
Mannino	2003	America	GOLD II	Healthy	878/8446	NA	NA	NA	316 ± 58	281 ± 47
Mannino	2003	America	GOLD III/IV	Healthy	228/8446	NA	NA	NA	340 ± 67	281 ± 47
Ferroni	1997	Europe	COPD	Healthy	33/16	9/18.8	68/58	NA	342 ± 61	233 ± 44

Data are expressed as cases/controls. Abbreviations: AECOPD, acute exacerbations of COPD; M/F, male/female; NA, not available.

### Quality assessment

Using the 9-star Newcastle–Ottawa Scale system, the total score of qualified case–control studies ranged from 6 to 9 stars (mean: 7.71; standard deviation: 0.76) (Supplementary Table S2).

### Overall analyses

**Figure 2:**
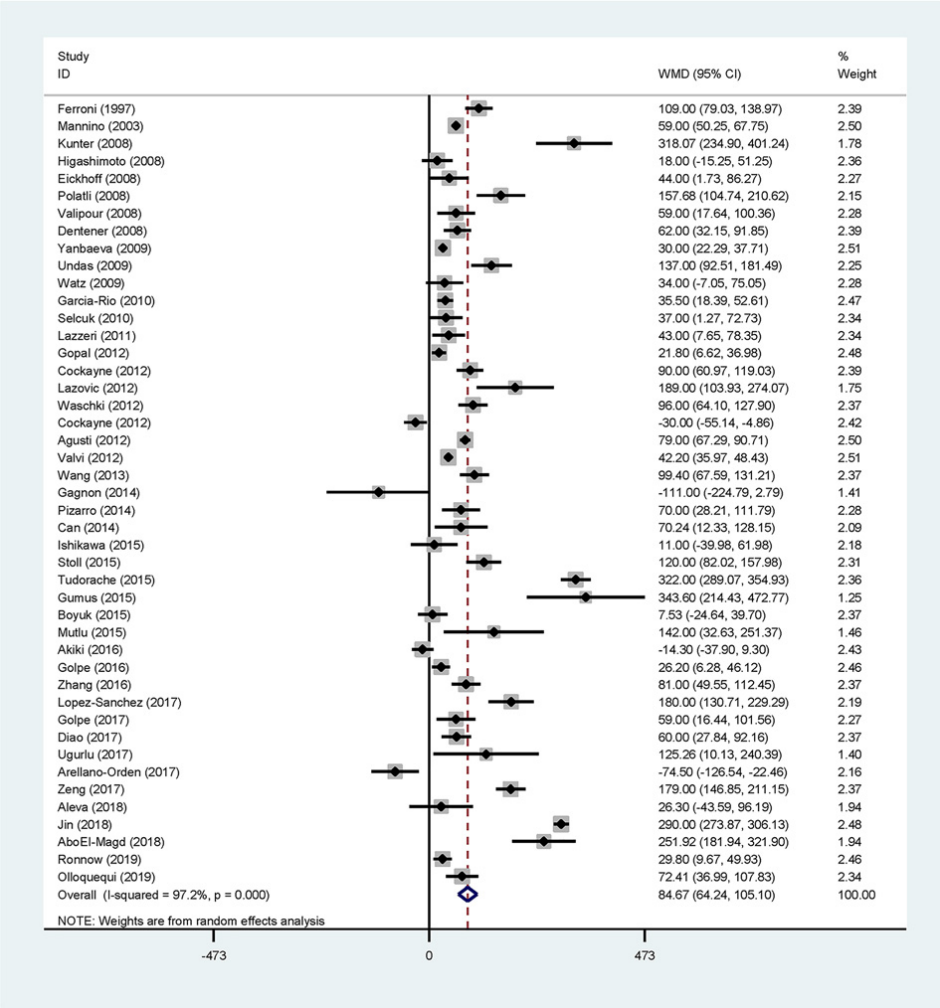
Forest plots for the comparisons of circulating fibrinogen between COPD patients and controls in overall analyses

Pooling the results of 44 qualified studies observed a significantly higher concentration of circulating fibrinogen in COPD patients than in controls (WMD: 84.67 mg/dl, 95% CI: 64.24–105.10, *P*<0.001), and there was strong evidence of between-study heterogeneity (*I*^2^: 97.2%, *P*<0.001) (Figure 2).

### Subgroup analyses

In view of the strong evidence of heterogeneity in overall analyses, it is necessary to explore possible causes by grouping studies according to COPD course, COPD severity, smoking habit in controls, sample size, and region, respectively.

**Figure 3:**
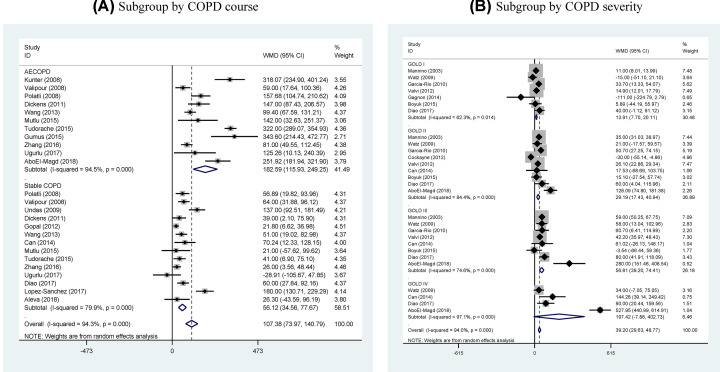
Forest plots for subgroup analyses Forest plots for subgroup analyses by COPD course (stable and acute exacerbations) (**A**) and by COPD severity (GOLD I, II, III, and IV) (**B**).

By COPD course (Figure 3A), the degree of increased circulating fibrinogen in patients with acute exacerbations of COPD (AECOPD) relative to controls (WMD: 182.59 mg/dl, 95% CI: 115.93–249.25, *P*<0.001, *I*^2^: 94.5%) tripled when compared with patients with stable COPD (WMD: 56.12 mg/dl, 95% CI: 34.56–77.67, *P*<0.001, *I*^2^: 79.9%). By COPD severity (Figure 3B), there was a graded increase in circulating fibrinogen concentration with the increased severity of COPD relative to respective controls (for GOLD I, WMD: 13.91 mg/dl, 95% CI: 7.70–20.11, *P*=0.014, *I*^2^: 62.3%; for GOLD II, WMD: 29.19 mg/dl, 95% CI: 17.43–40.94, *P*<0.001, *I*^2^: 84.4%; for GOLD III, WMD: 56.81 mg/dl, 95% CI: 39.20–74.41, *P*<0.001, *I*^2^: 74.6%; for GOLD IV, WMD: 197.42 mg/dl, 95% CI: −7.88 to 402.73, *P*<0.001, *I*^2^: 97.1%).

**Figure 4:**
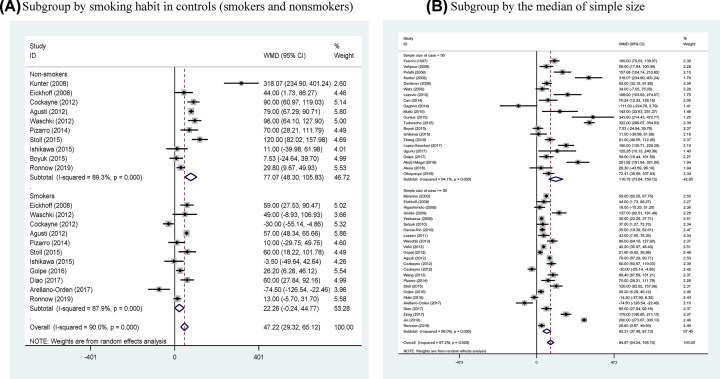
Forest plots for subgroup analyses Forest plots for subgroup analyses by smoking habit in controls (smokers and non-smokers) (**A**) and by sample size at the median value (sample size <50 and sample size ≥50) (**B**).

By smoking habit in controls (Figure 4A), the increase in circulating fibrinogen concentration was significantly greater in COPD patients relative to non-smoking controls (WMD: 77.07 mg/dl, 95% CI: 48.30–105.83, *P*<0.001, *I*^2^: 89.3%) than smoking controls (WMD: 22.26 mg/dl, 95% CI: −0.24 to 44.77, *P*<0.001, *I*^2^: 87.9%). By sample size, the difference in circulating fibrinogen concentration between COPD patients and controls was larger in studies with sample size <50 (the median value) (WMD: 116.78 mg/dl, 95% CI: 73.84–159.72, *P*<0.001, *I*^2^: 94.1%) than in studies with sample size ≥ 50 (WMD: 62.31 mg/dl, 95% CI: 37.49–87.13, *P*<0.001, *I*^2^: 98.0%) (Figure 4B).

Subgroup analyses by region are presented in Supplementary Figure S1.

### Meta-regression analyses

Other sources of heterogeneity were further explored through meta-regression analyses by modeling age, female percentage, and pack-year of COPD patients, and none of these factors reached statistical significance (all *P*>0.05) (Supplementary Figure S2).

### Cumulative and influential analyses

Cumulative analyses revealed no significant impact from first published study on subsequently studies in overall analyses (Supplementary Figure S3). In influential analyses, the impact of any single study on overall effect-size estimates was non-significant (Supplementary Figure S4).

### Publication bias

**Figure 5:**
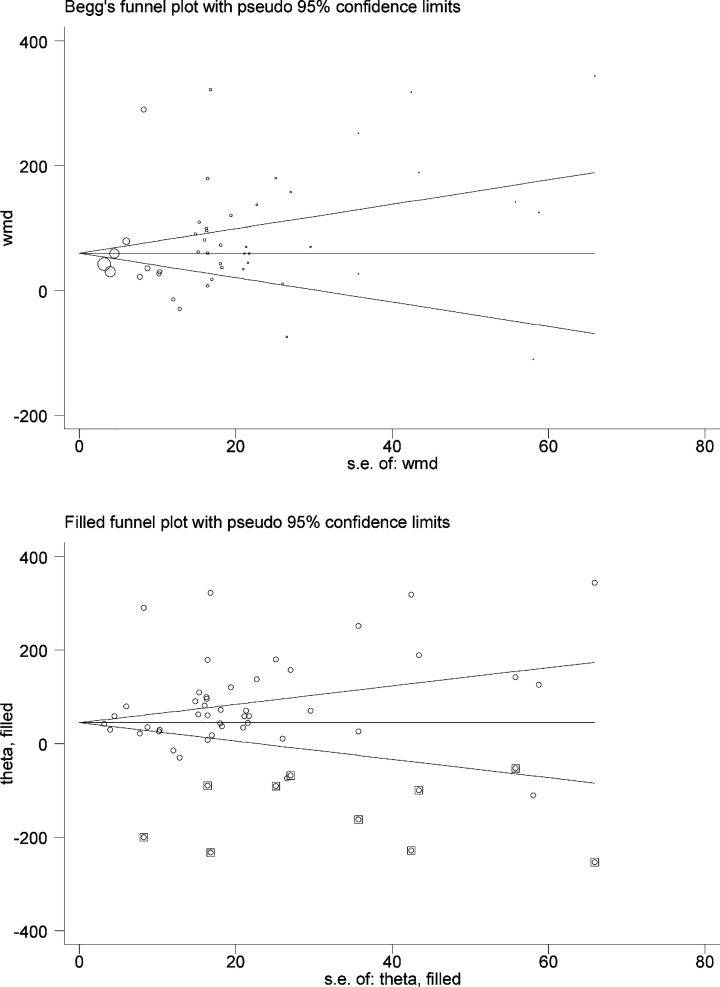
Begg’s and filled funnel plots in overall analyses Hollow circles represent all eligible studies, and solid squares represent potentially missing studies required to achieve symmetry.

Shown in Figure 5 are the Begg’s funnel plot and filled funnel plot in overall analyses. The Begg’s funnel plot seemed apparently symmetrical, and the Egger’s test indicated a low probability of publication bias (*P*=0.103). As reflected by filled funnel plot, there was an estimated ten missing studies required to make the Begg’s funnel plot symmetrical. After taking these ten missing studies into account, COPD patients still had a statistically significant higher concentration of circulating fibrinogen than controls (WMD: 46.30 mg/dl, 95% CI: 21.85–70.75, *P*<0.001).

## Discussion

The aim of this meta-analysis was to examine the association of circulating fibrinogen with COPD and its severity by pooling the results of 45 published articles. The key finding of this meta-analysis suggests that a graded, concentration-dependent, significant relation exists between higher circulating fibrinogen, and more severity of COPD. To the best of our knowledge, this is thus far the first meta-analysis that has evaluated the concentration-dependent association of circulating fibrinogen with COPD severity in the literature.

In human body, fibrinogen is mainly synthesized by the liver, and it is converted into fibrin by thrombin during blood coagulation [60]. Fibrinogen is a major acute-phase reactant, and its synthesis is up-regulated in response to inflammation [61], a major clinical feature of COPD. There is evidence that fibrinogen is implicated in clot formation and is associated with advanced COPD [11]. In addition, elevated fibrinogen in circulation was found to be associated with an increased risk of acute exacerbations in COPD [62]. Furthermore, as indicated by the nationally representative NHANES III data, impaired lung function is a correlate of fibrinogen concentration and the presence of high fibrinogen concentration increases mortality risk both in the overall population and among subjects with COPD [63]. It is hence reasonable to speculate that circulating fibrinogen is a promising clinical biomarker in predicting the risk and severity of COPD. Our findings based on a meta-analysis of 45 studies reinforced this speculation by showing that there is a graded, concentration-dependent, significant relation between higher concentration of circulating fibrinogen and more severity of COPD. It is also worth noting that statistical significance retained when analysis was restricted to studies with large sample sizes and when including theoretically missing studies as estimated by the fill and trim method to control publication bias, indicating the robustness of our meta-analytical findings.

Another important finding is that there is a possible interaction between circulating fibrinogen and cigarette smoking in our subgroup analyses by smoking habit in controls, as we interestingly noticed that the difference in circulating fibrinogen concentration was more obvious when compared with non-smoking controls than with smoking controls. Cigarette smoking is an established risk factor for the development of COPD, and it is of added interest to know whether the contribution of cigarette smoking to COPD is mediated by elevated fibrinogen in circulation, which cannot be reliably investigated in the present meta-analysis due to the unavailable individual participant data. We agree further explorations on the interaction or medication impact of cigarette smoking on circulating fibrinogen are needed.

The findings of this meta-analysis are susceptible to several possible limitations. First, language selection bias is possible, as we restricted literature search to the articles written in the English language. As estimated by McAuley and colleagues, the exclusion of gray literature from a meta-analysis may result in an overestimate of an association impact by an average of 12% [64]. Second, the majority of included studies in this meta-analysis are cross-sectional in design, which might yield recall bias and preclude comments on causality. Third, in our overall and subgroup analyses, the majority of comparisons were obsessed by moderate to high degree of between-study heterogeneity, which made it difficult to draw firm conclusions and required further explorations of other possible sources for heterogeneity. Fourth, nearly all qualified studies in this meta-analysis had circulating fibrinogen concentration measured only once, and did not evaluate its long-term change in the development of COPD. Fifth, the methods to assay circulating fibrinogen are not identical across studies, which might yield measurement bias. Thereby, the jury must refrain from drawing a conclusion until future large-scale, longitudinal, well-performed studies to confirm or refuse our findings.

Despite these limitations, our findings suggest a graded, concentration-dependent, significant relation between higher circulating fibrinogen and more severity of COPD. For practical reasons, circulating fibrinogen can be proposed as a promising biomarker and an early warning sign of the susceptibility to develop COPD in the future, as issued by the FDA [15], as well as a robust predictor for the severity of COPD. Importantly, circulating fibrinogen is an easy-to-assay biomarker and can be proposed as a more practical approach toward clinical translation applications.

## Supplementary Material

Supplementary Figures S1-S4 and Tables S1-S2

## Abbreviations

AECOPD: acute exacerbations of chronic obstructive pulmonary disease
COPD: chronic obstructive pulmonary disease
FDA: Food and Drug Administration
GOLD: Global Initiative for Obstructive Lung Disease
PRISMA: Preferred Reporting Items for Systematic reviews and Meta-Analyses
WMD: weighted mean difference
95% CI: confidence interval
